# Supplementary material for: The mwtab Python Library for RESTful Access and Enhanced Quality Control, Deposition, and Curation of the Metabolomics Workbench Data Repository
Source: Metabolites. 2021 Mar 12;11(3):163. doi: 10.3390/metabo11030163 (PMC8000456; doi:10.3390/metabo11030163)
Supplement: Supplementary file 1 [file metabolites-11-00163-s001.pdf]

Supplemental Table S1. List of analysis IDs and the format of files which could not be parsed into '~mwtab.mwtab.MWTabFile' objects.

File Format	Analysis ID
mwTab	AN000132, AN000152, AN000153, AN000154, AN000261, AN000418, AN000721, AN000956, AN001296, AN001297, AN001298, AN001299, AN001982, AN001996, AN001997, AN002105, AN002106, AN002107, AN002108, AN002149, AN002150, AN002209, AN002210, AN002235, AN002236, AN002237, AN002238, AN002239, AN002242, AN002255, AN002256, AN002257, AN002258, AN002259, AN002290, AN002291, AN002292, AN002298, AN002323, AN002324, AN002325, AN002326, AN002327, AN002328, AN002329, AN002330, AN002331, AN002347, AN002348, AN002363, AN002378, AN002379, AN002385, AN002386, AN002391, AN002392, AN002396, AN002397, AN002418, AN002420, AN002445, AN002446, AN002447, AN002448, AN002449, AN002450, AN002451, AN002453, AN002468, and AN002469
JSON	AN000020, AN000030, AN000041, AN000046, AN000047, AN000048, AN000049, AN000050, AN000051, AN000052, AN000053, AN000054, AN000055, AN000056, AN000057, AN000058, AN000059, AN000085, AN000086, AN000087, AN000094, AN000132, AN000152, AN000153, AN000154, AN000155, AN000156, AN000163, AN000167, AN000168, AN000171, AN000172, AN000226, AN000227, AN000228, AN000229, AN000230, AN000231, AN000233, AN000234, AN000237, AN000245, AN000246, AN000258, AN000259, AN000261, AN000270, AN000271, AN000274, AN000275, AN000284, AN000285, AN000317, AN000318, AN000332, AN000418, AN000856, AN000857, AN000858, AN000859, AN000860, AN000861, AN000937, AN000938, AN000945, AN000956, AN001311, AN001312, AN001313, AN001471, AN001517, AN001529, AN001530, AN001538, AN001539, AN001554, AN001555, AN001556, AN001557, AN001572, AN001573, AN001574, AN001575, AN001619, AN001624, AN001625, AN001626, AN001627, AN001647, AN001648, AN001651, AN001652, AN001653, AN001654, AN001655, AN001656, AN001657, AN001658, AN001681, AN001682, AN001683, AN001706, AN001721, AN001733, AN001738, AN001752, AN001763, AN001864, AN001865, AN002109, AN002172, AN002173, AN002197, AN002209, AN002210, AN002229, AN002230, AN002242, AN002290, AN002291, AN002292, AN002320, AN002334, AN002343, AN002347, AN002348, AN002393, AN002407, AN002408, AN002409, AN002418, AN002420, AN002445, AN002446, AN002447, AN002448, AN002449, AN002450, and AN002451
Both 'mwTab' and JSON	AN000132, AN000152, AN000153, AN000154, AN000261, AN000418, AN000956, AN002209, AN002210, AN002242, AN002290, AN002291, AN002292, AN002347, AN002348, AN002418, AN002420, AN002445, AN002446, AN002447, AN002448, AN002449, AN002450, and AN002451

Supplemental Table S2. List of common metabolite metadata headings from 'METABOLITES' blocks of MS analyses, Regular Expressions (RegExs) used to match field names, and examples of similar/matching fields present in analysis files.

<table>
<tr><th>Common Field Name</th><th>RegEx Pattern(s)</th><th>Example Matched Field Names</th></tr>
<tr><td>hmdb_id</td><td>r"(?i)[\s|\S]{,}(HMDB)"
r"(?i)(Human Metabolome D)[\S]{,}"</td><td>HMDB ID (*representative)
HMDB (*Representative ID)
HMDB_ID
HMDB ID (*Representative ID)
Representative HMDB ID
HMDB ID (*representative ID)
HMDBID
HMDB ID
Representative HMDB
Human Metabolome Database
HMDB.ID
HMDB
HMDB ID (*representativeID)
Human Metabolome DB</td></tr>
<tr><td>inchi_key</td><td>r"(?i)(inchi)[\S]{,}"</td><td>Inchi_Key
InChIKey
InchiKey
InChi key
InChi-Key
InChiKey
InChI Key
InchiKEY
INCHIKEY
InChI key</td></tr>
<tr><td>kegg_id</td><td>r"(?i)(kegg)$"
r"(?i)(kegg)(\s|_)(i)"</td><td>KEGG
KEGG I
Kegg ID
KEGG_ID
KEGG id
KEGG ID</td></tr>
<tr><td>moverz</td><td>r"(?i)(m/z)"</td><td>m/z
M/Z
m/z rounded</td></tr>
<tr><td>moverz_quant</td><td>r"(?i)(moverz)(\s|_)(quant)"
r"(?i)(quan)[\S]{,}(\s|_)(m)[\S]{,}(z)"</td><td>Quantified m/z
quantitated mz
Moverz Quant</td></tr>
</table>

<table>
<tr><td></td><td></td><td>quantified m/z
quantified mz
Quanitied m/z
quant_moverz
Quant m/z
quant mz
quantitated m/z</td></tr>
<tr><td>other_id</td><td>r"(?i)(other)(\s|_)(id)$"</td><td>Other ID
Other_ID</td></tr>
<tr><td>pubchem_id</td><td>r"(?i)(pubchem)[\S]{,}"</td><td>PubChem CID
Pubchem ID
PubChem
PubChem ID
PUBCHEM ID
Pubchem_Derivative
PubChem id
Pubchem Id
PUBCHEM</td></tr>
<tr><td>retention_index</td><td>r"(?i)(ri)$"
r"(?i)(ret)[\s|\S]{,}(index)"</td><td>retention time index
ri
Retention index
Retention Index
ret index
ret.index
retention index
retention index (min)
Ret. Index</td></tr>
<tr><td>retention_time</td><td>r"(?i)(r)[\s|\S]{,}(time)[\S]{,}"</td><td>retention_times
retention time index
Retention Time
Retention Times
Retention time
retention time(s)
rtimes(mins)
rtimes (min)
retention_time(min)
rtimes
Retention times
row retention time
Retention time (min)
Ret.Time
Retention Time(min)</td></tr>
</table>

		Retention Time (mins)<br>retention time<br>RTimes (min)<br>retention times<br>rtimes (mins)
